# Multifaceted Interactions Between Bile Acids, Their Receptors, and MASH: From Molecular Mechanisms to Clinical Therapeutics

**DOI:** 10.3390/molecules30153066

**Published:** 2025-07-22

**Authors:** Xuan Tang, Yuanjiao Zhou, Li Xia, Xiulian Lin, Yao Zhu, Menghan Chen, Jiayao Wang, Yamei Li

**Affiliations:** Key Laboratory for Quality Evaluation of Bulk Herbs of Hunan Province, School of Pharmacy, Hunan University of Chinese Medicine, Changsha 410208, China; 202204040121@stu.hnucm.edu.cn (X.T.); 202009030248@stu.hnucm.edu.cn (Y.Z.); 15376155784@163.com (L.X.); 20232047@stu.hnucm.edu.cn (X.L.); 202204090164@stu.hnucm.edu.cn (Y.Z.); 202304030101@stu.hnucm.edu.cn (M.C.); 11yyxf@stu.hnucm.edu.cn (J.W.)

**Keywords:** bile acid, bile acid receptor, MASH, metabolic disturbance

## Abstract

Metabolic dysfunction-associated steatohepatitis (MASH) represents a critical hepatic manifestation within the broader spectrum of metabolic syndrome. The pathogenesis of MASH is characterized by disruptions in lipid metabolism, inflammation, and fibrosis. Bile acids and their receptors are integral to the progression of MASH, primarily through their regulatory influence on the metabolic networks of the gut–liver axis. This review offers a comprehensive and systematic examination of the molecular mechanisms underlying bile acid biosynthesis, metabolic dysregulation, and receptor signaling anomalies in MASH. Furthermore, it explores the translational potential of these insights into clinical therapies. Bile acids and their receptors emerge as pivotal therapeutic targets for MASH. Future research should focus on an in-depth analysis of dynamic regulatory mechanisms and the optimization of multi-target combination therapies, thereby paving the way for significant clinical advancements.

## 1. Introduction

Metabolic dysfunction-associated steatohepatitis (MASH), previously referred to as non-alcoholic steatohepatitis (NASH), represents an advanced stage within the disease continuum of metabolic dysfunction-associated steatotic liver disease (MASLD) [1]. The primary pathological hallmark of MASH is the dysregulation of lipid metabolism within hepatocytes, resulting in the aberrant accumulation of triglycerides in the liver. This condition is further characterized by pathological processes such as inflammatory responses, cellular stress, and apoptosis [2,3]. The etiology of MASH is multifactorial, encompassing genetic predispositions, environmental influences (including dietary habits and obesity), and metabolic abnormalities (such as insulin resistance) [4]. In the fructose–palmitate–cholesterol-enriched diet mouse model, molecular studies revealed significant sex-dependent differences in hepatic parenchymal cell and hepatic stellate cell function, and similar sex-specific pathologies were observed in MASH patients. Key lipid metabolic pathways (e.g., fatty acid oxidation and cholesterol biosynthesis) were differentially regulated in female animals and female patients compared to males [5].

MASH is increasingly recognized as a significant global health concern, with epidemiological studies indicating a rising prevalence [6,7,8]. The progression of MASH can lead to a spectrum of severe complications, including liver fibrosis, cirrhosis, portal hypertension, and hepatocellular carcinoma [9]. Furthermore, there is a causal relationship between MASH and metabolic syndrome, which substantially elevates the risk of cardiovascular diseases, including coronary artery disease and myocardial infarction [10]. The current treatment modalities for MASH exhibit significant limitations in clinical management, characterized by a discernible disparity between expert guidelines and actual medical practice. Furthermore, there remains a global deficiency in standardized treatment protocols [11].

Bile acids (BAs) and their receptors serve a dual function in the onset and progression of MASH. They act as both contributors to metabolic disorders and as potential therapeutic targets [12]. This article integrates the mechanisms and clinical evidence of bile acids and their receptors with MASH, clarifies the efficacy and safety characteristics of existing treatment methods, and provides a framework for targeted therapy strategies and new drug development.

## 2. Methods

This article aims to comprehensively elucidate the complex network of actions of bile acids and their key receptors in MASH and deeply analyze their potential clinical implications. To achieve systematic evidence integration, we carefully designed and implemented a structured literature screening protocol. Initial searches were conducted in major biomedical literature databases, such as PubMed and Scopus. The search protocol was constructed based on core concepts (covering the target disease state, key molecules and signaling pathways, levels of action, core research areas, and therapeutic intervention strategies), and logical operators (AND, OR, NOT) were used to combine the search terms. The search covered all the relevant literature from the time of database establishment until July 17, 2025, with a focus on screening the latest research results published since 2020.

The research focuses on exploring the mechanisms and impacts of bile acids and their receptors in the development and progression of MASH. Specific research types include molecular mechanism experiments using in vitro systems or cell models (such as primary hepatocytes, Kupffer cells, hepatic stellate cell lines, intestinal organoids, and co-culture systems) to study bile acids or their receptors; studies using animal models (such as mice or rats induced by different diets or genetically modified through knockout or overexpression) to elucidate the pathophysiological processes of MASH or evaluate the effects of potential interventions; and clinical studies in MASH patients (such as cohort studies, case–control studies, cross-sectional surveys, and interventional clinical trials) [13]. In-depth analysis of the abnormal characteristics of bile acids metabolism in the MASH state, systematic examination of bile acids receptors (including their expression levels, functional states, and related signaling pathways, such as the farnesoid X receptor–fibroblast growth factor 15/19 (FXR-FGF15/19) pathway, TGR5 (the Takeda G protein receptor 5-)–cAMP (the 3′, 5′-cyclic adenosine monophosphate) pathway, and the pregnane X receptor/chimeric antigen receptor (PXR/CAR) regulation of drug-metabolizing enzymes in the specific roles they play in disease development and progression [14]. Additionally, the potential applications of agonists, antagonists, or modulators targeting specific bile acid receptors in MASH experimental models or patients are explored, including a deep molecular-level investigation of their mechanisms of action and a comprehensive evaluation of their therapeutic effects and safety.

In the literature search for experimental design, it is crucial to set up appropriate control groups. Basic research should include untreated cells or animals as controls. Clinical studies must establish corresponding control groups, such as placebo groups, standard treatment groups, or healthy individuals without MASH, to enable effective comparisons. Evaluation criteria cover multiple levels. At the molecular and mechanistic level, attention should be paid to changes in receptor expression or activity, alterations in signaling pathway-related molecules, effects of bile acid/receptor intervention on lipid/sugar metabolism, inflammation, oxidative stress, cell death (apoptosis/necrosis), gene and protein expression or function related to fibrosis processes, and changes in gut microbiota composition and barrier function [15]. In terms of clinical and preclinical efficacy, key indicators include the reduction or resolution of liver steatosis (confirmed by imaging or histology), decreased liver lobular inflammation (histological assessment), reduced hepatocyte ballooning (histological scoring), improvement or reversal of liver fibrosis stages (histopathology or non-invasive markers), changes in serum transaminase serum alanine aminotransferase (ALT/AST) levels, optimization of metabolic parameters, such as blood glucose and lipids, and alterations in body weight or composition [16]. Safety assessments should particularly document drug-related adverse events (with special attention to issues such as pruritus and increased low-density lipoprotein cholesterol associated with FXR agonists), analyze potential limitations in the mechanism of the intervention, and examine possible interactions with other drugs or pathways.

To obtain a comprehensive evidence chain from basic mechanisms to clinical value, a wide range of literature design types was included. This covered original basic research (in vitro experiments and animal studies) and original clinical research (randomized controlled trials, non-randomized controlled trials, cohort studies, case–control studies, and cross-sectional studies). Additionally, high-quality systematic reviews and meta-analyses evaluating the clinical effectiveness and safety of the drug, as well as high-level review articles providing important insights or concise summaries, were included. After completing the literature search, duplicate entries from different database search results were automatically removed using the literature management software Zotero 7.0.

## 3. Biosynthesis and Metabolism of Bile Acids

The synthesis and metabolism of bile acids is a core physiological process regulated by the human host in concert with its cohabiting gut microbiota [17]. This process encompasses the synthesis of bile acids in the liver, their subsequent modification in the gut by the microbiota, and the regulatory effects of the modified products on metabolic signaling pathways in the body. Dysregulation of this critical physiological process is closely associated with the development of MASH [18].

### 3.1. Synthesis Pathway of Bile Acids

Bile acids, as the principal constituents of bile, are derived from cholesterol [19]. Following their synthesis in the liver, these steroid acid compounds are predominantly stored in the gallbladder and subsequently released into the small intestine to fulfill their physiological roles as required [20]. Bile acid synthesis occurs via two primary pathways: the classical (neutral) pathway and the alternative (acidic) pathway. The classical pathway serves as the predominant route for bile acid synthesis, whereas the alternative pathway functions in a compensatory capacity in response to developmental, pathological, or metabolic demands.

#### 3.1.1. Classic Pathway (Neutral Pathway)

During hepatic metabolism, the biosynthesis of primary bile acids is initiated through a series of enzymatic transformations of cholesterol. The central regulatory mechanism of this metabolic pathway is significantly influenced by cholesterol 7-alpha hydroxylase (CYP7A1), which catalyzes the conversion of cholesterol to 7α-hydroxycholesterol [21]. This compound is subsequently transformed into two major bile acid components, cholic acid (CA) and chenodeoxycholic acid (CDCA), via a series of enzymatic reactions involving sterol 12α-hydroxylase (CYP8B1), sterol 27-hydroxylase (CYP27A1), and 3 beta-hydroxy steroid dehydrogenase type 7 (HSD3B7) [22]. The BA-FXRs signaling axis exerts a negative regulatory effect on CYP7A1 expression, establishing a critical network for maintaining metabolic homeostasis [23]. Hepatocyte nuclear factor 4α effectively enhances the transcriptional activity of the gene by binding to the CYP8B1 promoter region, thereby precisely modulating this response process [24].

#### 3.1.2. Alternative Pathway (Acid Pathway)

Alternative pathways constitute a critical mechanism for bile acid synthesis and are integral to liver disease, lipid metabolism, and host–microbial interactions. In instances where classical pathways are inhibited, such as through defects in CYP7A1 or excessive bile acid feedback inhibition, alternative pathways sustain bile acid synthesis by upregulating the expression of CYP27A1 as a compensatory mechanism. Within mitochondria, CYP27A1 is pivotal in these alternative pathways, facilitating the synthesis of non-12-hydroxylated primary bile acids by catalyzing the 27-hydroxylation of cholesterol [25]. In addition, it plays a role in the regulation of CDCA production, thereby providing a crucial metabolic branch for the maintenance of cholesterol homeostasis and the equilibrium of the bile acid pool [26,27].

The synthesis of bile acids is governed by complex regulatory networks at multiple levels, encompassing the transcriptional regulation of enzyme genes and the activation of signal transduction pathways. These regulatory elements achieve the precise regulation of metabolic pathways by establishing a dynamic equilibrium mechanism.

### 3.2. Bile Acid Metabolism

Bile acid metabolism constitutes a multifaceted physiological process encompassing binding and secretion, intestinal metabolism, and bacterial modification, as well as enterohepatic circulation. Within hepatocytes, bile acids conjugate with glycine or taurine via the enzyme bile acid CoA/amino acid N-acyltransferase (BAAT), resulting in conjugated bile acids, such as glycocholic acid and taurocholic acid [28]. This conjugation enhances their hydrophilicity and surface activity. The conjugated bile acids are subsequently secreted into the bile duct through the bile salt export pump (BSEP), stored in the gallbladder, and released into the duodenum postprandially to facilitate lipid emulsification [29].

MASH, the impairment of intestinal barrier function, and changes in the microbiome composition form a dynamic and causally interrelated cycle. Specifically, the abnormal structure of the gut microbiota can trigger damage to the mucosal barrier integrity; at the same time, impaired barrier function exacerbates the negative effects of microbiome dysbiosis, with both working in concert to promote the continuous deterioration of liver lesions. This core mechanism has been well validated in multiple scientific studies and holds significant guidance for the development of clinical strategies for the prevention and treatment of liver diseases [30].

Intestinal bacteria facilitate the debinding of bile acids through the action of bile salt hydrolase (BSH) and subsequently convert bile acids into deoxycholic acid (DCA) via 7α-dehydroxylation, as exemplified by the Hylemon–Björkhem pathway, resulting in the production of secondary bile acids [31]. Additionally, the bacterial flora can undergo modifications through oxidation, epimerization (such as the conversion between α and β configurations of hydroxyl groups), and esterification, thereby generating a diverse array of bile acid molecules [32]. The enterohepatic circulation of bile acids serves as a precise physiological regulatory mechanism, playing a crucial role in lipid metabolism as well as in the digestion and absorption processes. Once bile acid synthesis is complete, these compounds are secreted into the biliary system and temporarily stored in the gallbladder [33]. Upon lipid ingestion, the gallbladder contracts, releasing bile into the duodenum and jejunum, which facilitates the emulsification of fats and the absorption of fat-soluble nutrients [34,35]. The majority of bile acids are reabsorbed in the terminal ileum via specific transport mechanisms, such as the apical sodium-dependent bile acid transporter (ASBT), and subsequently return to the liver through the portal vein system. This process constitutes a fundamental component of enterohepatic circulation [36].

Studies have shown that abnormal increases in intestinal permeability and the destruction of epithelial cell junction complex structures, together with microbial community imbalance, constitute important mechanisms in the development of MASH. When the physical barrier function of the gut is compromised, microbial metabolites (including endotoxins, etc.) can enter the liver via the portal venous circulation, triggering local inflammatory responses and causing liver cell damage [37,38]. On the other hand, pathological changes in the composition of the gut microbiota (such as reduced abundance of beneficial bacteria and overgrowth of potential pathogenic bacteria), particularly metabolic functional abnormalities, such as decreased short-chain fatty acid synthesis, further promote the progression of MASLD to MASH [39].

The meticulous regulation of this metabolic network ensures that bile acids fulfill critical roles in digestion, metabolism, and immune regulation. Disruptions in any aspect of this network can lead to metabolic disorders. Aberrant binding and secretion of bile acids may result in cholestatic diseases, MASLD, diabetes, and obesity. Furthermore, altered intestinal metabolism and modifications in the gut microbiota may contribute to the development of inflammatory bowel disease, colorectal cancer, obesity, insulin resistance, metabolic syndrome, and atherosclerosis. Additionally, disturbances in the enterohepatic circulation can lead to MASLD, type 2 diabetes, obesity, metabolic syndrome, and dysregulated cholesterol metabolism [40,41].

## 4. The Relationship Between Bile Acids and Their Receptors in the Context of MASH

### 4.1. Bile Acid Receptors

Bile acid receptors are primarily categorized into two types: nuclear receptors and membrane receptors. The nuclear receptors encompass FXR, PXR, vitamin D receptor (VDR), and CAR. In contrast, the membrane receptors comprise TGR5and sphingosine 1-phosphate receptor 2 (S1PR2) [42].

As a pivotal regulator, FXR modulates lipid homeostasis via its various subtypes (such as FXRα1/α2) within the gut–liver axis [43]. PXR possesses a heterologous substance-sensing capability and orchestrates a gene regulatory network involved in the detoxification process [44]. Beyond its role in calcium–phosphorus metabolism regulation, VDR engages with bile acid signaling within the immune regulatory pathway [45]. CAR modulates bile acid and bilirubin metabolic networks by repressing CYP7A1 expression and maintains a coordinated regulatory relationship with the liver X receptor signaling pathway [46]. TGR5 is localized in metabolically active tissues and, upon activation, contributes to metabolic regulation by enhancing energy expenditure and metabolic efficiency [47]. S1PR2 influences the hepatic–intestinal axis signaling pathway, impacting inflammatory responses and fibrosis through multi-pathway interactions during the pathological progression of MASH [48,49].

Bile acids exert their biological effects by binding to the corresponding receptors. Among them, the major endogenous ligands of FXR are native bile acids. When these ligands bind to the ligand-binding domain of FXR, they induce conformational changes in the receptor, thereby activating its transcriptional regulatory activity. Activated FXR recognizes and binds to the FXR response element and recruits related coregulatory protein complexes. Known FXR ligands include CDCA and lithocholic acid [50,51]. In contrast, ligands for TGR5 include CDCA, DCA, and their taurine conjugates, such as taurochenodeoxycholic acid (TDCA) [52]. PXR primarily recognizes bile acid metabolites, such as 3-ketocholic acid and the acetylated derivatives of lithocholic acid. The ligand class of S1PR2 includes TDCA [53]. VDR is highly specific for lithocholic acid (LCA) [54]. CAR, although activated by bile acids, binds to specific bile acid isoforms that have not been fully elucidated to date [42].

The bile acid receptor system orchestrates physiological processes, including metabolic regulation, immune response, and inflammatory response, through a complex network of signaling mechanisms, thereby establishing a crucial molecular foundation for maintaining homeostasis (Table 1). Each receptor subtype exhibits synergistic and compensatory mechanisms, and research into their functions offers a theoretical basis for the treatment of MASH.

### 4.2. The Function of Bile Acids and Their Receptors in the MASH Pathway

In the pathological progression of MASH, aberrant bile acid metabolism is characterized by an excessive accumulation of toxic bile acids, including 12α-hydroxylated acid, alongside a diminished concentration of protective bile acids, such as ursodeoxycholic acid. This imbalance results in a distinctive disruption of the bile acid pool. Toxic bile acids have the capacity to inhibit the expression of downstream FXR target genes, thereby compromising bile acid homeostasis [79].

The metabolic network of bile acids is intricate, involving not only synthesis by the host but also regulation by the intestinal microbiota. The intestinal microbiota converts primary bile acids into secondary bile acids through dehydroxylation reactions [80]. Notably, a significant reduction in secondary bile acid levels has been observed in MASH mouse models. An imbalance in the intestinal microbial flora, such as an altered Firmicutes/Bacteroidetes ratio, leads to disorders in secondary bile acid production. This includes a reduction in the synthesis of non-12α-hydroxylated acids, such as porcine deoxycholic acid, which is closely associated with disease progression [81,82]. Furthermore, 3-succinylated cholic acid (3-sucCA) may exert a protective effect by regulating the intestinal flora. Specifically, 3-sucCA promotes the growth of the beneficial bacterium Akkermansia muciniphila, a process that enhances intestinal barrier function and reduces liver inflammatory response and fibrosis [31].

**Figure 1:**
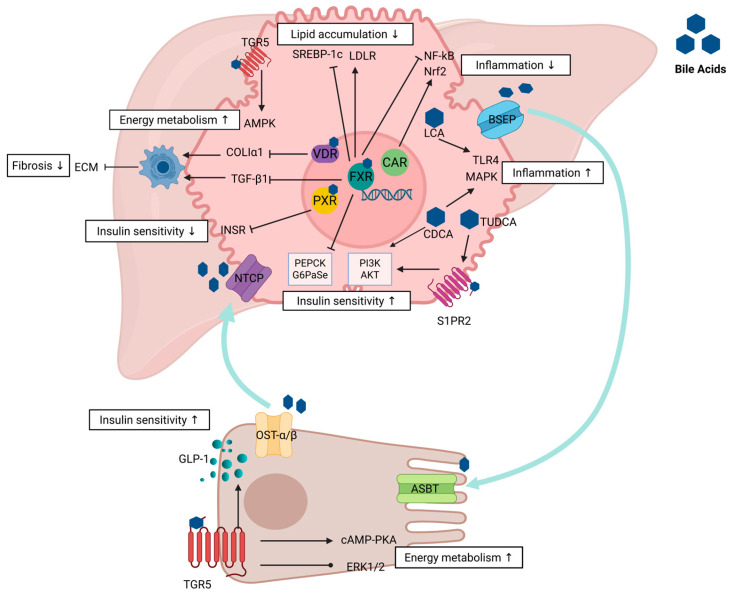
The pathological progression of MASH is progressive. As shown, FXR inhibits SREBP-1c expression and activates the low-density lipoprotein receptor (LDLR), thereby reducing abnormal lipid accumulation in the liver. When lipid accumulation in the healthy liver exceeds a certain threshold, it triggers an inflammatory cascade that further drives liver fibrosis. CAR and FXR exert anti-inflammatory effects by activating the Nrf2 pathway and inhibiting NF-kB signaling, respectively. In contrast, LCA and CDCA activate MAPK and TLR4 signaling pathways, respectively, and exacerbate inflammatory responses. FXR and VDR inhibit TGF-β1 and COLIα1, respectively. Energy metabolism and insulin sensitivity are inextricably linked to the process. FXR enhances insulin sensitivity by inhibiting PEPCK and G6Pase transcriptional activity, while CDCA and S1PR2 enhance insulin sensitivity by activating the PI3K/Akt pathway. In contrast, PXR decreases insulin sensitivity by inhibiting INSR signaling. In intestinal epithelial cells, TGR5 promotes GLP-1 secretion and enhances insulin sensitivity. In addition, TGR5 promotes energy consumption by activating the cAMP-PKA pathway and regulating extracellular signal-regulated kinases 1 and 2 (ERK1/2) activity. Bile acids synthesized from cholesterol in hepatocytes are actively transported into bile by BSEP. BA uptake from the intestine is mainly mediated by ASBT, while OSTα/OSTβ is responsible for BA export from the basolateral membrane to the portal vein. Hepatocytes re-uptake bile acids from portal vein blood through NTCP to complete hepatointestinal circulation. Overall, bile acids and their specific receptors play a central role in the pathological evolution of MASH by finely regulating lipid metabolism, inflammation, fibrotic processes, and energy homeostasis at multiple levels. Arrow: This refers to an increase in energy metabolism after receptors regulate pathways.

In the pathogenesis of MASH, the interplay between bile acids and cholesterol is characterized by a complex and detrimental cycle. Cholesterol serves as a direct precursor for bile acid synthesis, and its excessive accumulation triggers the classical synthesis pathway, specifically the CYP7A1-mediated 7α-hydroxylase pathway. However, this pathway frequently becomes dysregulated in pathological conditions [83]. The accumulation of cholesterol precursors and aberrant bile acids in the liver exerts direct cytotoxic effects, activating hepatic stellate cells and expediting the progression of fibrosis [84]. Furthermore, these substances can induce insulin resistance by interacting with receptors such as S1PR2, thereby contributing to systemic metabolic disturbances, including metabolic syndrome. Additionally, bile acids and their receptors play a critical role in liver metabolism, inflammatory responses, and the fibrotic process [85] (Figure 1).

#### 4.2.1. Regulation of Hepatic Lipid Accumulation and Inflammatory Processes

Bile acids exhibit multifaceted regulatory roles within the network of hepatic lipid metabolism and inflammation regulation. Their biological functions extend beyond the absorption and transport of lipids and lipid-soluble substances; they also engage in metabolic regulation and inflammatory response processes through the signaling pathways of various receptors [86].

The dysregulation of bile acid metabolism can result in impaired lipid absorption and hepatic lipid accumulation [19]. Bile acids are conveyed via hepatic–intestinal axis signaling involving FXR-FGF15/19, playing a crucial role in sustaining bile acid metabolic homeostasis and intestinal microbial diversity [87]. FXR enhances the expression of the low-density lipoprotein receptor (LDLR) to facilitate cholesterol clearance and suppresses the expression of sterol regulatory element-binding protein-1c (SREBP-1c) to decrease hepatic triglyceride production, thereby promoting fatty acid β-oxidation and mitigating hepatic steatosis [88]. Intestinal microbiota are involved in the conversion of primary bile acids, and the resultant secondary bile acids enhance the secretion of GLP-1 through the activation of TGR5 receptors, consequently mitigating the risk of hepatic lipid accumulation [89].

Bile acids demonstrate dual regulatory roles in the modulation of inflammation. Under physiological conditions, they exert anti-inflammatory effects by inhibiting immune cell infiltration, modulating neutrophil activity, and downregulating the expression of genes associated with inflammation. Conversely, under pathological conditions, an imbalance in bile acid metabolism may precipitate proinflammatory responses. Elevated levels of hydrophobic bile acids, such as CDCA, can compromise mitochondrial function, induce apoptosis in hepatocytes, and concurrently activate the mitogen-activated protein kinase (MAPK) and phosphatidylinositol-3 kinase/protein kinase B (PI3K/Akt) pathways, thereby facilitating the progression from steatosis to MASH. Furthermore, dysbiosis-induced alterations in secondary bile acid ratios, such as the accumulation of LCA, may activate the Toll-like receptor 4 (TLR4) signaling pathway in Kupffer cells, exacerbating hepatic inflammatory damage [89].

The regulation of macrophage function by bile acids depends on the activation process of specific receptors, with TGR5 being a typical example. The activation signal of this receptor can induce macrophages to differentiate into an anti-inflammatory M2 phenotype, thereby inhibiting the secretion of proinflammatory factors and significantly reducing liver inflammation. In the pathogenesis of MASH, this mechanism can effectively alleviate the progression of the disease [90]. For example, ursodeoxycholic acid can enhance the activation of TGR5, prompting macrophages to shift towards an M2 polarized state, thereby controlling inflammatory responses. Additionally, dynamic changes in the bile acid spectrum (such as increased concentrations of primary bile acids) also participate in regulating the infiltration and accumulation of macrophages derived from monocytes in the liver. Specifically, in an animal model of MASH induced by a high-fat/high-cholesterol/taurine sodium diet, certain bile acids (such as CA) play a role in regulating the structure of the gut microbiota and the composition of the bile acid spectrum, further delaying the recruitment of macrophages in liver tissue and ultimately slowing the progression of MASH pathology [91,92].

Bile acids exert indirect effects on natural killer (NK) cell activity. For instance, alterations in the composition of bile acids (such as an increase in primary bile acid concentrations) have been found to correlate with the recruitment process of natural killer T (NKT) cells within the liver. Specific primary bile acids (such as α-muricholic acid) can induce an increase in the secretion of CXCL16 protein by sinusoidal endothelial cells, a process that promotes NKT cell-dependent immune responses; this chain reaction ultimately may constitute a regulatory pathway that mediates NK cell functional activation [93].

Bile acid regulatory networks encompass a variety of receptors and signaling pathways, each contributing distinctively to the modulation of inflammatory responses. FXR and TGR5 are instrumental in attenuating the activity of transcription factors, notably NF-kB, thereby diminishing the synthesis of proinflammatory cytokines, including tumor necrosis factor-α (TNF-α) and interleukin-6 (IL-6), among others [86,94]. Furthermore, TGR5 modulates inflammation through additional mechanisms. Specifically, TGR5 activation initiates the cAMP-dependent protein kinase A (PKA) signaling pathway, which subsequently inhibits the assembly of inflammasome complexes and curtails the secretion of pivotal inflammatory mediators, such as interleukin-1β (IL-1β) [95]. Conversely, TGR5 contributes to the amelioration of the hepatic inflammatory microenvironment by facilitating the secretion of GLP-1 in intestinal endocrine cells. Activation of PXR may attenuate inflammatory responses through inhibition of the NF-kB pathway; however, prolonged activation could potentially exacerbate inflammation by promoting lipid accumulation. Activation of CAR can suppress the expression of proinflammatory cytokines, such as interleukin-1β (IL-1β) and interleukin-6 (IL-6), in Kupffer cells and mitigate oxidative stress-related inflammation by upregulating pathways, like nuclear factor erythroid 2-related factor 2 (Nrf2). Furthermore, the activation of S1PR2 can enhance the secretion of proinflammatory factors via the mitogen-activated protein kinase (MAPK) and NF-kB pathways, whereas inhibition of S1PR2 can alleviate inflammatory damage associated with alcoholic liver disease [96].

#### 4.2.2. The Relationship Between Bile Acids and the Progression of Liver Fibrosis

Patients diagnosed with MASH exhibited notable disturbances in bile acid metabolism. This was evidenced by elevated concentrations of total bile acids in both serum and hepatic tissues. Specifically, bile acids known for their hepatotoxic properties, such as DCA and LCA, were found to be abnormally increased and demonstrated a strong correlation with the severity of liver fibrosis [97]. Conversely, ursodeoxycholic acid, which is recognized for its hepatoprotective effects, displayed a declining trend [98,99].

The regulation of bile acid metabolism homeostasis is a critical determinant in the progression of pathological conditions [100]. Notably, during the fibrotic process, there is a marked reduction in the expression levels of metabolic enzymes associated with solute carrier family 27 member 5 (SLC27A5). This reduction facilitates the accumulation of toxic bile acids, subsequently exacerbating the inflammatory response and fibrosis through the dual activation of the NF-kB and transforming growth factor-β (TGF-β) signaling pathways [101]. Additionally, bile acids are capable of modulating related signaling pathways via the synergistic interaction of nuclear and membrane receptors. Using TDCA and glycochenodeoxycholic acid (GDCA) as exemplars, these compounds activate the ERK1/2 pathway via TGR5 receptors, thereby promoting the proliferation of HSCs and the expression of genes associated with fibrosis. Targeted inhibition of this pathway has been shown to effectively mitigate the fibrotic phenotype [97]. Furthermore, the molecular mechanisms by which the intestinal microbiota influences the fibrosis process through secondary bile acid metabolism, such as DCA, are of significant interest. The dysbiosis-induced abnormal accumulation of DCA can activate HSCs and exacerbate the inflammatory response. Conversely, specific bioactive substances, such as Antarctic krill peptides, have the potential to modulate the bile acid profile and inhibit fibrosis progression through microbiota reconstruction [102,103].

The activation state of FXR is intricately linked to the amelioration of fibrosis associated with MASH. FXR modulates the expression of the *CYP7A1* and *CYP8B1* genes, thereby influencing the anabolic balance of bile acids and lipid homeostasis, which are critically associated with liver fibrosis [104]. Furthermore, FXR inhibits the activation of hepatic stellate cells by modulating the activity of the NOD-like receptor protein 3 (NLRP3) inflammasome, thus impeding the molecular progression of liver fibrosis [105,106]. Additionally, FXR directly targets hepatic stellate cells to inhibit the transforming growth factor-beta 1 (TGF-β1)/Smad signaling pathway, resulting in reduced expression of alpha-smooth muscle actin (α-SMA) and type I collagen, thereby exerting anti-fibrotic effects.

As a ligand-dependent transcription factor, VDR forms a complex with the Retinoid X Receptor (RXR) and inhibits the expression of collagen genes, such as Collagen 1A1 (COL1A1), by binding to the vitamin D response element (VDRE). This binding action impedes the transformation of HSCs into myofibroblasts [107]. Conversely, the activation of CAR may exacerbate the risk of fibrosis by promoting hepatocyte proliferation, particularly in the context of chronic injury. Nevertheless, CAR’s anti-inflammatory and metabolic regulatory functions continue to present potential therapeutic avenues. Additionally, S1PR2 is highly expressed in HSCs, and its activation enhances the proliferation, migration, and contractile capabilities of HSCs by integrating bile acid signaling and facilitating cell–cell interactions, thereby promoting extracellular matrix (ECM) secretion.

#### 4.2.3. Regulation of Energy Metabolism and Modulation of Insulin Sensitivity

Bile acids facilitate fatty acid oxidation through various mechanisms, including nuclear receptor signaling, regulation of mitochondrial function, interaction with gut microbiota, and mitigation of oxidative stress. These processes collectively reduce lipotoxicity and enhance energy metabolism efficiency, thereby improving insulin sensitivity in a systematic manner [108]. The evidence from animal models with FXR knockout demonstrates a typical phenotype of glucose metabolism disorder, underscoring the essential role of this receptor in maintaining glucose homeostasis [109]. Furthermore, activation of FXR has been shown to upregulate the expression of thermogenic genes in adipose tissue, thereby promoting energy expenditure [110]. At the level of energy metabolism regulation, the BA-TGR5 signaling axis exhibits distinct metabolic regulatory properties. TGR5 activation initiates the cAMP-PKA signaling pathway, which regulates energy metabolism by enhancing the thermogenic function of brown adipose tissue and improving glucose uptake efficiency in skeletal muscles, thereby effectively ameliorating insulin resistance [111]. Notably, skeletal muscle serves as the primary tissue for metabolism. The enhancement of insulin sensitivity can lead to a reduction in hepatic glucose output and alleviate the metabolic burden imposed by hyperglycemia on liver function, thereby ameliorating glycolipid metabolic disorders associated with MASH. TGR5 facilitates mitochondrial biosynthesis by activating the AMP-activated protein kinase (AMPK) pathway, thereby improving the efficiency of ATP production. Furthermore, TGR5 plays a pivotal role in regulating the body’s energy metabolism through various signaling pathways, including AKT and ERK1/2. Alterations in the intestinal microbiota can influence bile acid synthesis, subsequently impacting metabolic processes. By modulating the composition of the intestinal microbiome, the bioavailability of non-12α-hydroxylated bile acids, such as ursodeoxycholic acid (UDCA), can be increased, thereby enhancing the thermogenic effects mediated by TGR5 [112].

Activation of the TGR5 receptor has been shown to stimulate the secretion of GLP-1 by intestinal L cells. GLP-1 not only enhances the functionality of pancreatic islet β-cells but also facilitates glucose uptake in peripheral tissues [113,114]. TGR5 facilitates the phenotypic transformation of cell secretion from glucagon to GLP-1 by upregulating the expression of prohormone convertase 1 (PC1) in pancreatic islet alpha cells. From an inflammation regulation perspective, TGR5-mediated inhibition of the NF-kB signaling pathway can substantially decrease the expression levels of proinflammatory cytokines, such as tumor necrosis factor-alpha (TNF-α) and interleukin-6 (IL-6), thereby mitigating insulin resistance associated with chronic inflammation [66,115]. Furthermore, activation of FXR can significantly suppress the transcriptional activities of phosphoenolpyruvate carboxykinase (PEPCK) and glucose-6-phosphatase (G6Pase), thereby effectively reducing the rate of hepatic gluconeogenesis. As a downstream effector molecule of the farnesoid X receptor (FXR), fibroblast growth factor 19 (FGF19) plays a crucial role in the precise regulation of metabolic homeostasis through dual mechanisms: the inhibition of CYP7A1 expression and the enhancement of hepatic glycogen synthesis [116,117]. The activation of PXR may adversely affect insulin sensitivity by inhibiting insulin receptor (INSR) signaling. Tauroursodeoxycholic acid (TUDCA) has been shown to activate the S1PR2 signaling pathway, thereby enhancing the activity of the insulin-degrading enzyme (IDE) and effectively ameliorating hyperinsulinemia and insulin sensitivity [70]. Upon binding to its bile acid-derived ligands, S1PR2 potentiates the PI3K/Akt insulin-signaling axis, thereby enhancing peripheral glucose uptake and improving insulin sensitivity, indicating that this receptor–ligand axis constitutes a promising therapeutic target for insulin resistance. In states of insulin resistance, the accumulation of cholesterol metabolism intermediates, such as 26-hydroxycholesterol, can lead to mitochondrial dysfunction, resulting in an inadequate energy supply and other related complications [118].

## 5. Bile Acids Are Anticipated to Serve as a Therapeutic Target for the Management of MASH

### 5.1. Intervention in the Bile Acid Pathway as a Therapeutic Approach for Managing MASH

#### 5.1.1. Investigation into the Molecular Mechanisms Underlying the Regulation of Bile Acid Synthesis by Pharmaceuticals

At the molecular regulation level, targeted interventions in bile acid synthesis pathways have demonstrated significant potential. Recent advancements in the development of novel treatment strategies have been noteworthy. The application of ASBT inhibitors, such as GSK233072, has been shown to effectively decrease the reabsorption of intestinal bile acids, thereby promoting the conversion of hepatic cholesterol into bile acids, accelerating cholesterol metabolism, and reducing hepatic fat accumulation [119]. By interfering with the enzyme systems responsible for the conversion of cholesterol to bile acids [120], or through the administration of pharmacological agents, such as UDCA/TUDCA, to modulate the bile acid pool, these approaches can effectively mitigate liver damage induced by bile acid toxicity [121]. By modulating the expression levels of key enzymes such as CYP7A1 and CYP27A1 and enhancing the activity of oxysterol 7α-hydroxylase (CYP7B1), the therapeutic efficacy of obeticholic acid (OCA) can be collectively improved [122,123]. FXR induces fibroblast growth factor 19 (FGF19), which primarily activates the fibroblast growth factor receptor 4 (FGFR4) and β-Klotho signaling pathways via the ERK1/2 signaling cascade, thereby inhibiting *CYP7A1* gene transcription and reducing bile acid synthesis. Concurrently, the peroxisome proliferator-activated receptor delta (PPARδ) agonist achieves negative regulation of bile acid synthesis by inhibiting the transcriptional process of CYP7A1 [124].

From the perspective of microbial–host interactions, probiotic YTP preparations enhance BSH activity by modulating bacterial functions, as indicated in [125,126]. Consequently, this modulation leads to a reduction in hepatic lipid deposition and inhibits the progression of MASH, as noted in [82]. Inhibition of BSH activity can decrease the production of secondary bile acids, thereby reducing the hepatic load and toxicity associated with bile acids. The process of 7α-dehydroxylation in the liver is a critical component of secondary bile acid synthesis, and its inhibition may diminish the accumulation of toxic bile acids, as suggested by the authors of [80].

**Figure 2:**
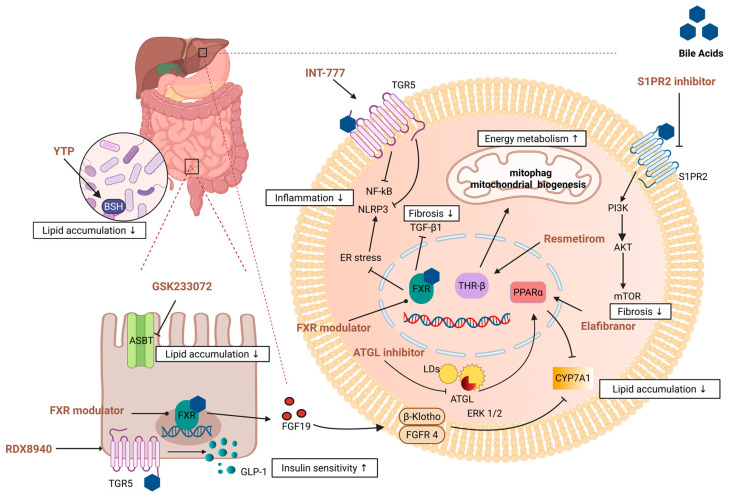
A variety of drug targets exert therapeutic effects on MASH by modulating key signaling pathways. In terms of lipid metabolism regulation, FXR agonists induce FGF19 production, which, in turn, activates the FGFR4/β-Klotho complex primarily via the ERK1/2 pathway and ultimately inhibits CYP7A1 transcriptional activity. ATGL and Elafibranor also downregulate CYP7A1 expression by activating PPARα receptors. These mechanisms work together to reduce hepatic lipid deposition. In addition, GSK233072 alleviates lipid accumulation by blocking the bile acid transporter ASBT. YTP reduces lipid accumulation in the liver by altering intestinal microbial function and enhancing BSH activity. At the inflammatory regulatory level, the bile acid receptors TGR5 and FXR both inhibit the activation of the inflammatory body NLRP3, thereby alleviating the inflammatory response. For example, INT-777, as an agonist of TGR5, achieves anti-inflammatory effects by inhibiting NF-kB signaling. TGR5 activation also promotes GLP-1 secretion and enhances insulin sensitivity, as shown by RDX8940. FXR activation inhibits the expression of the profibrotic factor TGF-β1 in liver fibrosis, while S1PR2 inhibitors mitigate fibrosis by targeting S1PR2 receptors and inhibiting downstream PI3K/AKT/mTOR signaling pathways. In terms of energy metabolism enhancement, Resmetirom, as a THR-β selective agonist, promotes mitochondrial autophagy and enhances mitochondrial biosynthesis, thereby optimizing energy metabolism efficiency. These drugs show good potential in MASH therapy by synergistically improving liver–gut axis function and comprehensively regulating liver inflammation and lipid metabolism homeostasis. This mechanism of action not only exploits the metabolic properties of intestinal flora but also promotes the repair of liver tissue and inhibits the damage process by intervening in specific molecular signaling networks. Arrow: This means that TGR5 promotes GLP-1 secretion, which increases insulin sensitivity.

The translocator protein (TSPO) ligand, Atriol, has been shown to mitigate the excessive production of reactive oxygen species (ROS), inhibit the overproduction of bile acids, and effectively suppress ROS-dependent inflammatory signals, such as NLRP3, as well as fibrosis-related pathways, including TGF-β. This mechanism collectively leads to the downregulation of chemokine (C-X-C motif) ligand 1 (CXCL1) expression, thereby ameliorating pathological conditions such as liver steatosis, inflammation, and fibrosis [127]. Furthermore, the overexpression of Forkhead Box A3 (FOXA3) in the liver has been demonstrated to effectively prevent diet-induced MASH by activating the TGR5 signaling pathway. FOXA3 plays a crucial role in regulating lipid metabolism by promoting lipolysis and inhibiting the expression of genes associated with bile acid absorption, resulting in elevated plasma bile acid levels. This regulatory function not only contributes to reductions in body weight and fat content but also improves liver indices, decreases plasma aminotransferase levels, and significantly reduces hepatic triglyceride accumulation and the extent of liver fibrosis [128] (Figure 2).

#### 5.1.2. Strategies for Drug Research Focusing on Bile Acid Metabolism

The receptors within the bile acid signaling system hold significant biological importance in the domain of metabolic regulation. Through physiological mechanisms such as bile acid metabolism, glycolipid homeostasis regulation, and immune response modulation, these receptors have emerged as critical targets for intervention in metabolic disorders and inflammatory conditions.

FXR agonists are categorized into several types, including bile acid derivatives, non-bile acid-derived steroid FXR agonists, nonsteroidal FXR agonists, and partial FXR agonists [129]. Notably, FXR agonists, exemplified by OCA, have demonstrated efficacy in ameliorating liver fibrosis (with improvements of at least one grade) [130] and reducing steatosis levels by enhancing insulin sensitivity and modulating the bile acid–glucose–lipid metabolism axis [131,132]. Beyond its role in metabolic regulation, FXR is integral to maintaining intestinal circulation integrity. Specific agonists can effectively prevent dysfunction of the intestinal barrier and bacterial translocation in cholestasis [133]. Additionally, FXR antagonists, such as glycine-β-muricholic acid (Gly-MCA), can indirectly enhance the composition of the bile acid pool by improving the interaction between intestinal microbiota and bile acids [134]. Nevertheless, systemic activation of the FXR can lead to adverse effects, including pruritus, dyslipidemia, and disruptions in intestinal microbiota [135,136]. Consequently, the current research is directed towards the development of specific FXR modulators, such as Gly-MCA, and selective FXR modulators, such as selective bile acid receptor modulators (SBARMs), with the objective of optimizing the therapeutic window and minimizing systemic side effects [137,138]. The pharmacokinetic parameters of XZP-5610, as predicted by the physiologically based pharmacokinetics (PBPK) model, exhibit a high degree of concordance with clinical data, particularly in terms of liver concentration predictions. This characteristic is critical for drugs targeting hepatic conditions, such as MASH, and may enhance their local anti-inflammatory and anti-fibrotic efficacy [139]. The development of novel nonsteroidal FXR agonists, such as Tropifexor, seeks to optimize receptor selectivity and substantially mitigate adverse reactions, such as pruritus, associated with traditional OCA drugs, while preserving their anti-fibrotic and metabolic regulatory effects [140,141].

TGR5 serves as a crucial regulatory component within the bile acid system [142]. Its activation in the intestinal tract enhances glycolipid metabolism by promoting GLP-1 secretion, while intrahepatic TGR5 activation may mitigate inflammatory responses through inhibition of the NF-kB pathway [143]. Therapeutic agents targeting TGR5 not only address liver metabolic disorders but also exert beneficial effects on MASH-related cardiovascular abnormalities, indicating their protective properties across multiple systems [144]. Various agonists, such as UDCA and TLCA, exhibit distinct effects in oncological studies, suggesting the presence of a bidirectional regulatory mechanism in cancer processes [47]. Furthermore, activation of the TGR5-FGF15/19 axis can ameliorate insulin resistance, although it may impact the absorption of fat-soluble vitamins [145]. The dual receptor agonist INT-767, which synergistically activates the FXR/TGR5 signaling pathway, demonstrates a multifaceted ameliorative effect on hepatic steatosis, inflammatory damage, and fibrosis progression in animal models, offering novel insights for the combined treatment of MASH [146].

Recent studies have provided groundbreaking insights into the molecular mechanisms underlying the action of PXR antagonists [147], thereby establishing a theoretical basis for the development of targeted PXR modulators, such as partial agonists or antagonists, which can therapeutically restore bile acid homeostasis. While VDR agonists have demonstrated potential in ameliorating insulin resistance and reducing hepatic lipid accumulation, there remains a need for precise regulation of their metabolic effects and the associated risk of bile acid imbalance [148]. S1PR2 inhibitors exhibit substantial clinical potential. By inhibiting the PI3K/AKT/mTOR signaling pathway, they not only impede the progression of hepatocellular carcinoma but also concurrently modulate abnormal lipid metabolism and fibrosis development. It is imperative for current research to systematically assess the safety and clinical efficacy of various bile acid receptor modulators through standardized clinical trials, with particular emphasis on the pivotal regulatory role of S1PR2 antagonists in the progression of MASLD-HCC [149].

Chelating agents, including cholestyramine, colestipol, and colesevelam, impede intestinal circulation by binding to bile acids within the intestinal tract. This process accelerates the conversion of cholesterol to bile acids, thereby reducing serum low-density lipoprotein cholesterol levels and mitigating hepatic inflammatory damage [150]. Targeting the bile acid metabolism network, molecular docking is used for the rapid screening of multi - target combinations of natural triterpenes to identify and optimize MASH candidate drugs cost - effectively and with high throughput, followed by validation in vitro and in vivo. Inhibitors of adipose triglyceride lipase (ATGL) attenuate ATGL activity, which diminishes the signaling of peroxisome proliferator-activated receptor α (PPARα) and facilitates the conversion of bile acids into hydrophilic bile acids. Activation of PPARα can suppress the expression of CYP7A1, consequently reducing bile acid synthesis. This transformation process can disrupt the absorption of dietary lipids and ameliorate metabolic disorders [151]. Although Elafibranor, a dual agonist of PPARα/δ, has demonstrated efficacy in modulating lipid metabolism and inflammatory response in clinical trials, its long-term efficacy and safety require further validation [152]. The activation of the NAMPT/NAD + /SIRT1 signaling pathway has been shown to optimize bile acid composition and decrease the production of proinflammatory cytokines, thereby ameliorating metabolic disorders in MASH model animals [123,153]. Resmetirom, a selective agonist of the thyroid hormone receptor-β (THR-β), demonstrates therapeutic potential by modulating hepatic and systemic lipid metabolism [154]. It facilitates the breakdown of both exogenous and endogenous fats into free fatty acids through the regulation of thyroid hormone signaling pathways. Additionally, Resmetirom enhances mitochondrial biogenesis and autophagy, thereby increasing energy expenditure.

### 5.2. The Clinical Application of Bile Acids and Receptor Modulators in the Treatment of MASH

The clinical management of MASH adheres to a stepwise therapeutic approach. The foundational treatment strategy emphasizes lifestyle modifications, particularly targeting weight management and exercise interventions. For patients with a pathological diagnosis of MASH who do not have diabetes, a combination therapy of pioglitazone and vitamin E may be considered a treatment option [155]. In the realm of adjunctive therapies, both orlistat and bariatric surgery have demonstrated clinical efficacy [156].

The exploration of bile acid receptor modulators in the treatment of MASH represents a promising avenue of research. Bile acids, through their interaction with specific receptors, perform a crucial regulatory role in maintaining lipid metabolic homeostasis, modulating energy balance, and regulating inflammatory responses and programmed cell death.

Based on preclinical research data, the FXR agonist OCA demonstrates a multifaceted pharmacological profile. A phase II clinical trial of OCA confirmed its efficacy in ameliorating liver fibrosis and reducing inflammatory responses. However, the trial also identified potential adverse events, including pruritus and dyslipidemia [157]. The inadequate therapeutic response observed in some patients may be attributed to the CYP7B1-mediated activation of alternative bile acid synthesis pathways [123]. Phase III clinical trials further substantiated OCA’s efficacy in improving liver fibrosis, although they did not achieve statistically significant differences in the resolution of MASH pathology. Additionally, there is a need to consider the potential cardiovascular risks associated with long-term OCA administration [158].

Given the limitations associated with OCA, significant advancements have been achieved in the research and development of novel nonsteroidal FXR agonists. Through the optimization of receptor selectivity and pharmacokinetic properties, these compounds have demonstrated improved safety profiles in phase II clinical trials [159]. Concurrently, TGR5 receptor agonists remain in the nascent stages of research and development, necessitating further empirical evidence to substantiate their efficacy and safety [94]. The TGR5 agonist RDX8940 operates by stimulating GLP-1 secretion and enhancing insulin sensitivity without impeding gallbladder emptying [160]. Notably, the dual FXR/TGR5 agonist INT-767 has exhibited promising anti-fibrotic, anti-inflammatory, and metabolic regulatory effects in preclinical studies [148,161]. Pharmacological investigations indicate that low-dose administration can satisfy treatment window requirements, potentially reducing the incidence of clinical adverse reactions. Long-term efficacy data suggest that this compound offers sustained therapeutic benefits, presenting new strategic options for the comprehensive management of MASH [146].

Clinical studies have demonstrated that bile acid chelators exhibit significant efficacy in modulating insulin sensitivity and mitigating lipid accumulation in hepatocytes [162]. In the development of CYP7A1/CYP27A1-targeted therapeutics, it is imperative to consider the impact of gender-related variables on pharmacokinetics and therapeutic outcomes. In a phase IIb double-blind controlled study of MASH, the Elafibranor treatment group exhibited notable histopathological improvements, with a significantly higher proportion of MASH remission compared to the placebo group. Additionally, the average reduction in fibrosis stage was 0.65 ± 0.61, in contrast to an increase of 0.10 ± 0.98 observed in the placebo group [152]. In a phase III clinical trial informed by prior phase II data, Elafibranor was evaluated using histopathological response and fibrosis improvement as dual endpoints [163]. However, the recent research indicates variability in the drug’s efficacy, potentially linked to genetic differences among patients, such as the activation of S100A4 and epithelial–mesenchymal transition (EMT) pathways [164]. While ASBT inhibitors have the potential to mitigate liver bile acid accumulation by modulating enterohepatic circulation, thereby ameliorating metabolic abnormalities and fibrosis, they have been associated with adverse gastrointestinal effects, such as diarrhea [150,159]. Currently, combined therapeutic strategies, including the synergistic use of OCA and GLP-1 receptor agonists, which address mechanisms related to improving insulin resistance and inhibiting fibrosis progression, are in the preliminary stages of clinical trials [26,158].

At the molecular signaling network level, the regulatory network comprising signal molecules, such as FXR, PPARα, and S1P, plays a pivotal role in controlling lipotoxicity, inflammatory responses, and the fibrosis process. Intestinal FXR antagonists and sphingolipid metabolic enzyme inhibitors, developed based on these insights, have progressed to the preclinical validation phase [165] (Table 2).

## 6. Discussion

The diagnosis of MASH still primarily relies on tissue pathology examination. However, given the invasive nature and associated risks of liver biopsy, one of the current research priorities is to advance the development and application of non-invasive diagnostic technologies. This will significantly improve the convenience of MASH diagnosis and reduce the burden on patients. In terms of treating MASH, the core focus is on identifying potential molecular targets, designing effective drug intervention strategies, and implementing comprehensive disease management approaches, with the aim of preventing disease progression, liver cirrhosis, and related adverse events [12].

Research on the treatment of MASH continues to advance, with novel therapeutic strategies and potential drug targets consistently emerging [175]. At the diagnostic technology level, metabolomics is employed to detect dynamic changes in active lipid components in patients, thereby providing biomarker support for early screening and intervention [176]. Current clinical research is centered on multiple mechanisms of action, with GLP-1 receptor agonists potentially contributing to the amelioration of the pathological processes associated with metabolic-related fatty liver [177]. In the realm of traditional Chinese medicine, Yinchenwuling Powder (YCWLP) has been utilized in the clinical treatment of MASH; however, the multi-component synergistic mechanisms of this compound require systematic analysis [178]. Concurrently, FGF19/21 analogs have demonstrated significant anti-steatosis, anti-inflammatory, and anti-fibrosis effects in various experimental models [179]. Targeted interventions in the transcriptional activity of the CAR gene regulation mechanism and hypoxia-inducible factor 2α (HIF-2α) offer new research directions for the development of gender-specific treatment strategies [176,180].

The research framework utilizing “C. elegans” as a model organism has demonstrated distinct advantages in the study of bile acid signal transduction and the analysis of lifespan regulation mechanisms. The findings from this research could provide significant insights into the identification of novel therapeutic targets [181]. In the context of microbiome intervention strategies, the dysregulation of intestinal flora homeostasis is closely associated with the progression of MASH [182]. The current research focuses on reestablishing the function of the liver–gut axis through the supplementation of probiotics, microbial transplantation, or the modulation of microbial metabolites [183,184]. The experimental drug LPJZ-658 has been shown to ameliorate MASH-related bacterial imbalances by modulating serum bile acid levels, thereby elucidating a clear interaction between bile acids and the microbiome [185]. In the realm of precision medicine, advancements in gene editing technologies targeting genetic susceptibility genes, such as patatin-like phospholipase domain-containing 3 (PNPLA3) and 17-beta hydroxysteroid dehydrogenase 13 (HSD17B13) [186], alongside immunomodulation and hepatocyte regeneration therapies utilizing mesenchymal stem cells and their secretion of extracellular vesicles (EVs), represent a novel therapeutic approach for the reversal of liver fibrosis [183]. Furthermore, ongoing research into the liver immune microenvironment, particularly the dynamic regulatory mechanisms of natural killer cells within the context of MASH pathology, is progressively unveiling new opportunities for therapeutic intervention [187].

Enhancing physical activity or modifying dietary patterns, such as increasing walnut consumption, can effectively enhance the biological activity of adiponectin. This, in turn, optimizes the hepatic lipid metabolism pathway and reduces lipid accumulation in hepatocytes [188]. Factors including elevated body mass index (BMI), glycemic imbalance, and insulin resistance can exacerbate disease progression [189]. It is advisable to develop a dynamic assessment system based on patients’ clinical presentations and implement a stratified management plan for individuals with metabolic disorders, such as diabetes and cardiovascular diseases. For patients with advanced fibrosis, the use of targeted anti-fibrotic drugs is particularly recommended [190,191]. Recent studies have integrated digital pathology technology with artificial intelligence algorithms, offering a novel technical approach to analyzing the mechanisms underlying treatment-induced fibrosis regression [192].

This article focuses on the role of bile acids and their receptors in MASH pathology, covering signal transduction pathways and gene expression regulation, clarifying their central role in disease initiation and progression. By integrating clinical trials and basic research evidence, this article further verifies the role of these molecules in MASH and its related complications. (e.g., liver disease and cardiovascular disease), providing novel perspectives for disease prevention and clinical intervention strategies. Based on the multiple mechanisms of action described above, this study explores the potential application of bile acids and their receptors in MASH therapy, aiming to facilitate innovative drug development and optimization of existing therapies [193].

In conclusion, the intricate pathological processes of MASH are intricately linked to the bile acid metabolism network and its receptor regulation. Although the current study has elucidated the molecular mechanisms by which bile acid dysregulation exacerbates liver damage through multiple receptor pathways, the translation and application of related therapeutic strategies remain significantly challenging [162]. The efficacy and progress in drug development have yet to meet clinical expectations. Challenges related to drug safety and tolerance, metabolic comorbidities, and systemic impacts persist as major concerns. It is imperative to continue exploring novel therapeutic approaches. Future research should focus on constructing dynamic regulatory models, elucidating receptor collaboration mechanisms, and concurrently advancing the development of new drugs and the design of individualized combination treatment plans. This will provide a theoretical foundation and practical direction for overcoming the challenges associated with MASH treatment.

## Figures and Tables

**Table 1:** The role of bile acid receptors in maintaining physiological homeostasis.

Function	Effect	Receptor	References
Maintenance of bile acid homeostasis	Reduction of bile acid synthesis: inhibition of CYP7A1 expression through post-transcriptional mRNA degradation, small heterodimer partner (SHP)-mediated transcriptional inhibition, and the entero-hepatic FGF15/19 signaling pathway, thereby reducing bile acid synthesis	FXR	[55,56]
Anti-inflammatory	Inhibition of nuclear factor-kappa B (NF-kB): inhibition of the activation of the NF-kB pathway by direct binding to NF-kB components or the regulation of their upstream kinases (e.g., IKBKG and IκBα)	FXR	[57]
	The inhibition of NF-κB activity via cAMP-PKA or AKT signaling pathway reduces the production of proinflammatory factors such as TNF-α and IL-6	TGR5	[35,58]
	Inhibits M1 polarization of macrophages and promotes M2 polarization and directly modulates macrophage immune responses	PXR	[59]
Regulates enterohepatic circulation	Upregulation of ASBT expression promotes intestinal reabsorption of bile acids	FXR	[60]
	Inhibition of NTCP expression reduces bile acid hepatic uptake		[61]
Reduces serum HDL cholesterol	Upregulation of SR-BI enhances the efficiency of SR-BI-mediated selective cholesterol uptake by HDL	FXR	[62,63]
Intervenes in hepatic fibrosis	Inhibits hepatic stellate cell (HSC) activation by directly regulating gene expression in HSCs and indirect mechanisms (such as reducing hepatocyte injury, regulating bile acids, and inflammatory signals)	FXR	[64,65]
Regulates glucose metabolism	Increases intracellular cAMP level through Gsα protein, promotes calcium influx into L cells, and triggers glucagon-like peptide-1(GLP-1) secretion	TGR5	[66,67]
	The activation of PI3K/Akt upregulates GLUT4 transport to cell membranes, enhancing glucose uptake by peripheral tissues	FXR	[68,69]
Improves insulin sensitivity	Indirectly enhances IDE expression or activity by regulating the S1P metabolic pathway, thereby optimizing insulin clearance and signaling	S1PR2	[70]
	Inhibition of SREBP-1c by SHP and reduced levels or activity of SREBP-1c helps alleviate insulin resistance caused by lipid oversynthesis and accumulation	FXR	[71]
Increases energy consumption	Increased mitochondrial activity via D2-mediated local thyroid hormone production and PGC1α increase	TGR5	[72]
Detoxification	Promotes LCA hydroxylation, which converts LCA to less toxic products (e.g., UDCA or MDCA) and enhances conjugation metabolism	VDR	[73]
	CYP3A4 and CYP2B are activated to accelerate the metabolism of xenobiotics and improve the clearance rate of toxic substances.	PXR	[74]
	Upregulates the inducible phase I enzyme (CYP3A) and upregulates the phase II enzymes (UGT, SULT, and GST), forming a complete detoxification chain of “metabolic-binding-excretion”		[75,76]
	After upregulating MRP2 and MRP3, they act as efflux pumps to promote the discharge of toxins, drugs, or endogenous metabolites into the intestinal cavity or blood, reducing their accumulation in the body	CAR	[77,78]

**Table 2:** Clinical application of bile acids and receptor modulators in MASH treatment.

Category	Compound	Status	Effect	References
FXR regulator	OCA	Phase III	Improves liver fibrosis but may induce skin itching and dyslipidemia	[166]
	Tropifexor	Phase II	The proportional area of collagen is significantly reduced	[167]
	XZP-5610	Phase I	Improves liver histology	[139]
	Gly-MCA	Preclinical	Improves liver lipid accumulation, endoplasmic reticulum stress, and inflammatory response	[168]
	MET 409	Phase III	Reduces liver fat content	[169]
TGR5 agonist	RDX8940	Preclinical	Enhances insulin sensitivity	[160]
	INT-777	Preclinical	Inhibits liver inflammation and fibrosis	[170]
S1PR2 inhibitor	JTE-013	Preclinical	Reduces liver damage and fibrosis	[171]
Double agonist	INT-767	Preclinical	Regulates metabolism; anti-inflammatory; anti-fibrosis	[148,161]
	Elafibranor	Phase III	Anti-fibrosis	[164]
Bile acid pool regulator	ASBT inhibitor	Preclinical	Lower serum cholesterol	[172]
	Resmetirom	Phase III	Reduces TG and LDL-C	[173]
			Relieves liver fibrosis	[154]
	norUDCA	Phase II	Lower serum ALT	[174]

## Data Availability

Data are contained within the article.

## Abbreviations

The following abbreviations are used in this manuscript:

BAs	Bile acids
MASH	Metabolic dysfunction-associated steatohepatitis
MASLD	Metabolic dysfunction-associated steatotic liver disease
CA	Cholic acid
CDCA	Chenodeoxycholic acid
DCA	Deoxycholic acid
LCA	Lithocholic acid
UDCA	Ursodeoxycholic acid
CYP7A1	Cholesterol 7 alpha-hydroxylase
CYP8B1	Sterol 12α-hydroxylase
CYP27A1	Sterol 27-hydroxylase
CYP7B1	Oxysterol 7α-hydroxylase
NF-kB	Nuclear factor kappa B
ASBT	Apical sodium-dependent bile acid transporter
BSH	Bile salt hydrolase
3-sucCA	3-succinylated cholic acid
ERK1/2	Extracellular signal-regulated kinases 1 and 2
Gly-MCA	Glycine-β-muricholic acid
BSEP	Bile salt export pump
TDCA	Taurochenodeoxycholic acid
SHP	Small heterodimer partner
FXR	Farnesoid X receptor
PXR	Pregnane X receptor
VDR	Vitamin D receptor
CAR	Chimeric antigen receptor
TGR5	Takeda G protein receptor 5
S1PR2	Sphingosine 1-phosphate receptor 2
GLP-1	Glucagon-like peptide-1
HSCs	Hepatic stellate cells
OCA	Obeticholic acid
HCC	Hepatocellular carcinoma
